# Transcriptome and metabolome analysis reveals key genes and secondary metabolites of *Casuarina equisetifolia* ssp. *incana* in response to drought stress

**DOI:** 10.1186/s12870-023-04206-x

**Published:** 2023-04-18

**Authors:** Shike Zhang, Chunmei He, Long Wei, Shuguang Jian, Nan Liu

**Affiliations:** 1 CAS Engineering Laboratory for Vegetation Ecosystem Restoration on Islands and Coastal Zones, South China Botanical Garden, Chinese Academy of Sciences, Guangzhou, 510650 China; 2 College of Life Sciences, Gannan Normal University, Ganzhou, 341000 China; 3 University of Chinese Academy of Sciences, Beijing, 100049 China; 4 Guangdong Provincial Key Laboratory of Silviculture, Protection and Utilization, Guangdong Academy of Forestry, Guangzhou, 510520 China

**Keywords:** *Casuarina equisetifolia*, Drought stress, Transcriptome, Metabolome

## Abstract

*Casuarina equisetifolia* is drought tolerant, salt tolerant, and able to grow in barren environments. It is often used to reduce wind damage, to prevent sand erosion, and to help establish plant communities in tropical and subtropical coastal zones. To determine the basis for its drought tolerance, we conducted transcriptomic and metabolic analyses of young branchlets under a non-drought treatment (D_0h) and 2-, 12-, and 24-h-long drought treatments (D_2h, D_12h, and D_24h). A total of 5033 and 8159 differentially expressed genes (DEGs) were identified in D_2h/D_0h and D_24h/D_0h. These DEGs were involved in plant hormone signal transduction, jasmonic acid (JA) biosynthesis, flavonoid biosynthesis, and phenylpropanoid biosynthesis. A total of 148 and 168 differentially accumulated metabolites (DAMs) were identified in D_12h/D_0h and D_24h/D_0h, which were mainly amino acids, phenolic acids, and flavonoids. In conclusion, *C. equisetifolia* responds to drought by regulating plant hormone signal transduction and the biosynthesis of JA, flavonoid, and phenylpropanoid. These results increase the understanding of drought tolerance in *C. equisetifolia* at both transcriptional and metabolic levels and provide new insights into coastal vegetation reconstruction and management.

**Supplementary Information:**

The online version contains supplementary material available at 10.1186/s12870-023-04206-x.

## Introduction

Drought is a common abiotic stress affecting plant growth and development [1]. Many physiological and biochemical processes of plants are affected by drought stress, resulting in reactive oxygen species accumulation, membrane structure damage, ion imbalance, and inhibition of enzyme activity, photosynthesis, and respiration [2, 3]. Plants have evolved various strategies to adapt to drought stress, such as morphological, cell physiological, metabolic, and molecular changes [4, 5]. As global climate change has increased the frequency of drought, the mechanisms of plant responses or tolerance to drought have attracted extensive attention of researchers [6, 7].

Plants have the ability to adapt to environmental stress by regulating physiological, molecular, and metabolic processes [3]. To reduce the osmotic and oxidative stress caused by drought, plants accumulate osmotic-regulating substances (e.g., soluble sugar and proline), promote the activity of protective enzymes (e.g., superoxide dismutase, catalase, and peroxidase) and increase the content of non-enzyme free radical scavengers (e.g., reduced glutathione and vitamin C) [8, 9]. Phytohormones help plants to improve their adaptability to drought. The abscisic acid (ABA)-mediated signaling pathway induces stomatal closure and regulates the drought responsive genes, which can improve plant drought tolerance [10, 11]. Jasmonate (JA) also mediates the response of plants to drought stress and participates in the biosynthesis of secondary metabolites involved in defense [12–14].

With the development of high-throughput sequencing technology, several recent studies have used RNA sequencing (RNA-seq) to reveal the expression patterns and signal regulation pathways of drought stress-related genes, and to identify the key genes and molecular mechanisms of plant responses to drought stress [1, 15]. In *Tamarix psammophila*, for example, researchers found 1618 and 2716 differentially expressed genes (DEGs) after 1 and 2 weeks of drought treatment, respectively; these DEGs were mainly involved in the mitogen-activated protein kinase (MAPK) signaling pathway, tryptophan and α-linolenic acid metabolism, and biosynthesis of flavonoid and phenylpropanoid [1]. Analysis of drought-stressed *Seriphidium transiliense* seedlings by the weighted gene co-expression network (WGCNA) method indicated that transcription factors mainly belonging to WRKY, bHLH, NAC, LEA, AP2/ERF, MYB, GRAS, C2H2, MADS, and bZIP families were important in the response to drought [11].

Complementary to transcriptomics, metabolomic analysis can reveal how the synthesis, decomposition, and transformation of metabolites change in response to external environmental stress [1, 16]. For example, the contents of lipids and organic acids in *T. psammophila* increased under drought [1]. *Arabidopsis* responded to drought stress by accumulating flavonoids, amino acids, and lipids [17, 18]. The important metabolites differing in drought-tolerant vs. drought-sensitive sesame plants under drought stress were ABA, amino acids, and organic acids [19]. Metabolomic analysis showed that phenylalanine, oxidized glutathione, and ABA were related to the drought tolerance of *Sophora davidii* [6]. Moreover, the biosynthesis and accumulation of unsaturated fatty acids were found to be involved in the response of maize and peanut to drought stress [20, 21].

*Casuarina equisetifolia* is a pioneer tree species commonly used for wind prevention and sand fixation in Australia, the Pacific Islands, tropical America and Southeast Asia [22, 23]. *C. equisetifolia* has strong drought and barren tolerance, therefore it can grow in impoverished and arid coastal zones [24]. Previous studies showed that the maximal photochemical efficiency and the activity of ribulose bisphosphate carboxylase oxygenase of *C. equisetifolia* were not affected by water stress [25, 26]. *C. equisetifolia* could adapt to nutrient deficiency and drought stress by increasing the ratios of total phenolics : nitrogen and extractable condensed tannins : nitrogen [27]. However, the molecular mechanisms of *C. equisetifolia* responses to drought stress are still unknown. In this study, we used transcriptomic and metabolomic analyses to study the drought response of *C. equisetifolia* branchlets. We identified several key genes and metabolic pathways involved in the drought response of *C. equisetifolia.* This study thereby increases our understanding of the mechanisms of *C. equisetifolia* response to drought and may also provide useful information for the management and restoration of zonal vegetation.

## Materials and methods

### Plant materials and experimental design

*C. equisetifolia* ssp. *incana* was selected for this study, because it has high drought, wind, and salt tolerance, and has been widely planted in southeast coastal areas of China [23]. The seedlings of *C. equisetifolia* were preserved and cultivated in South China Botanical Garden, Chinese Academy of Sciences (Tianhe District, Guangzhou, Guangdong Province, China). After the germination of *C. equisetifolia* seeds in a 1:1 (v/v) mixture of vermiculite and perlite, its seedlings were transferred to plastic pots (17.5 cm inner diameter and 17.5 cm height; 2 seedlings/pot) containing a 3:1 (v/v) mixture of sand and coconut coir. The seedlings with a height of 20 cm were used in this study. The used vermiculite, perlite, sand, and coconut coir were sterilized by gamma ray irradiation. As not all seedlings form mycorrhizal or nodule with fungi or *Frankia*, sterilization was carried in order to ensure that all seedlings grow under uniform biological medium environment. In addition, this study mainly focused on the drought resistance of *C. equisetifolia* excluding the contribution of root symbioses [28].

Drought treatment was implemented by controlling irrigation. The volume water content (VWC) of the medium was checked three times a day. The control and drought treatment corresponded to 45% and 10% respectively. The soil moisture sensor (Hydrosense II) equipped with a CS659 probe (Campbell Scientific, USA) was used to determine the VWC of the medium [29]. The branchlets of *C. equisetifolia* seedlings were collected at four time points: when the VWC reached 45% as the control (D_0h), and respectively 2 h (D_2h), 12 h (D_12h) and 24 h (D_24h) after the VWC reached 10% as the drought treatment. The time course of previous studies on tolerance of halophytes is within several hours (0–48 h), which is conducive to revealing the response of plants to abiotic stress at the gene level [30, 31]. *C. equisetifolia* has been proved to be excellent in drought tolerance and can make rapid response to environmental stress [32]. Moreover, our preliminary experiment (tested by quantitative real-time PCR, qRT-PCR) showed that that 2 and 24 h were sensitive time points for *C. equisetifolia* to respond to drought stress. Thus, in order to understand its molecular response to drought stress, the time course of this study refers to the previous studies of halophytes. There were three biological repetitions at each stage. After the branchlets of the *C. equisetifolia* seedlings were collected, they were immediately frozen in liquid nitrogen and stored at -80 ℃ for RNA extraction and metabolite profiling.

### RNA extraction and sequencing

The total RNA of branchlets was extracted by TRIzol (Invitrogen, USA), and the genomic DNA was removed by DNase I (Takara, Japan). The quality of RNA samples was evaluated with a 2100 Bioanalyser (Agilent, USA) and a NanoDrop ND-2000 (Thermo Fisher Scientific, USA). The RNA samples meeting the quality criteria (OD_260 / 280_ = 1.8–2.2, OD _260 / 230_ ≥ 2.0, RIN ≥ 6.5, 28 S: 18 S ≥ 1.0, > 2 µg) were used to construct libraries and sequences. Sequencing was completed at Shanghai Majorbio Bio-pharm Co., Ltd. (http://www.majorbio.com/) using an Illumina NovaSeq 6000 System (Illumina, USA). SepPrep (https://github.com/jstjohn/SeqPrep) and Sickle (https://github.com/najoshi/sickle) software were used for the quality control of the raw data. The data were analyzed on a Major Bio Cloud Platform (https://cloud.majorbio.com/), and the reference genome version was fafu_v1 (http://forestry.fafu.edu.cn/db/Casuarinaceae/index.php). Fragments per kilobase per million (FPKM) was used to normalize and calculate gene expression levels. Raw counts were analyzed for DEGs using DESeq2 based on a negative binomial distribution with *P*-adjust < 0.05 and |log_2_FC| ≥ 1. Gene ontology enrichment was analyzed by Goatools (https://github.com/tanghaibao/goatools) and the Fisher test with *P*-adjust < 0.05. The National Center for Biotechnology Information (NCBI), Non-redundant (Nr, http://ftp.ncbi.nih.gov/blast/db/FASTA/nr.gz), Kyoto Encyclopedia of Genes and Genomes (KEGG, https://www.kegg.jp/), and Gene Ontology (GO, http://geneontology.org/) databases were used for gene function annotation.

### Metabolome extraction and analysis

The metabolites were extracted and analyzed by Wuhan Metware Co., Ltd. (https://www.metware.cn/). The freeze-dried branchlets were ground at 30 Hz in a mixing mill MM400 (Retsch, Germany) for 15 min. The powder (100 mg) was extracted overnight with 1 ml of 70% methanol (v/v) at 4 °C. After centrifugation at 10,000 *g* for 10 min, each supernatant was absorbed (Cnwbond Carbon-GCB SPE Cartridge, Anpel, China) and passed through a 0.22-µm pore size filter. The extracted samples were collected and subjected to metabolome analysis by an UPLC system (Shim-pack UFLC Shimadzu CBM30A, Shimadzu, Japan) and a tandem mass spectrometry (MS/MS) system (Applied Biosystems 6500 Q TRAP, Thermo Fisher Scientific, USA). The working parameters of the UPLC and MS were the method as described by [33]. Based on the Metware database, metabolites were identified according to secondary spectrum information, and metabolites were quantified by multi reaction monitoring of triple quadrupole mass spectrometry. Metabolite abundance was quantified according to peak area. The following thresholds were used to determine whether metabolites differed among treatments (D_0h, D_12h, and D_24h): variable importance in projection (VIP) ≥ 1, *P*-value < 0.05, fold-change ≥ 2, and fold-change ≤ 0.5]

### Quantitative real-time PCR analysis

The remaining RNA samples (D_0h, D_12h, and D_24h) from RNA-seq experiment were used for qRT-PCR analysis. According to the manufacturer’s instructions, the cDNAs were synthesized by the GoScript^TM^ Reverse Transcription System (Promega, USA). The qRT-PCR was performed with the Unique Aptamer^TM^ qPCR SYBR® Green Master Mix (Novogene, China) in a LightCycler® 480 II real-time PCR system (Roche, Switzerland). The PCR cycling condition was 95 °C for 2 min, 40 cycles of 95 °C for 15 s, 60 °C for 1 min, 95 °C for 15 s, 60 °C for 1 min, 95 °C for 15 s, and the final extension was 60 °C for 15 s [34]. The *elongation factor 1-alpha* (*EF1α*) and *ubiquitin* (*UBI*) were used as reference genes [35, 36]. The expression level of the target gene in each sample was calculated by the 2^−ΔΔCT^ method [37]. The expression level of each gene in all samples (D_0h, D_2h, and D_24h) was set to 1 in the control (D_0h). Three biological and technical replicates were performed for qRT-PCR. The qRT-PCR primers were designed by the Integrated DNA Technologies (http://www.idtdna.com/Primerquest/Home), and were listed in Table S1.

## Results

### Transcriptome of C. equisetifolia in response to drought stress

To determine the molecular response of *C. equisetifolia* to drought, we performed transcriptome sequencing of branchlets collected at three time points (0, 2, and 24 h after drought treatment) with three independent biological replicates for each time point. A total of 403,360,560 clean reads with GC content ranging from 47.15 to 47.88% were generated from RNA-seq, including 131,801,006; 130,745,702; and 140,813,852 clean reads from 0, 2, and 24 h, respectively. The Q30 base percentage of each library was > 93%, indicating that high-quality reads were obtained and could be used for further analysis. An average of 94.67% of clean reads could be mapped to the reference genome, and > 82% of the clean reads from each library were uniquely mapped reads (Table S2 ).

**Fig. 1:**
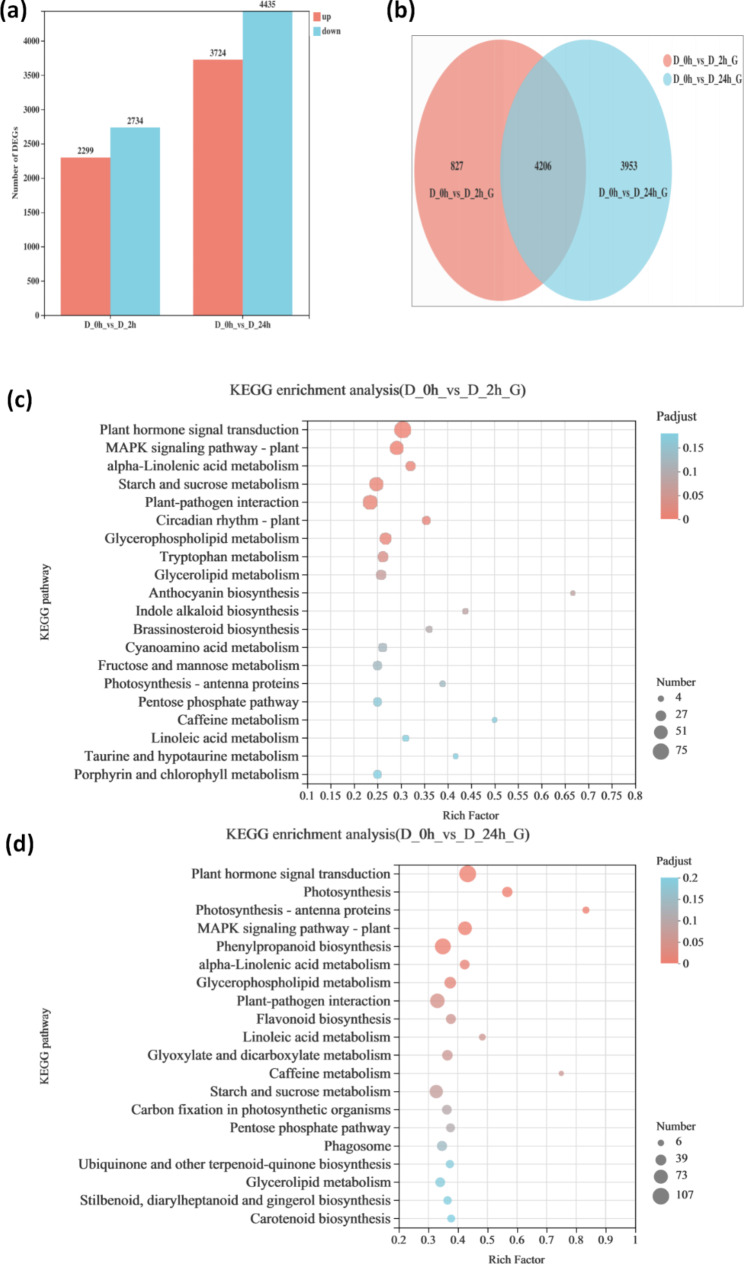
Numbers of DEGs and the main KEGG pathways enriched by DEGs in *C. equisetifolia* leaves that were exposed to drought stress for 2 h (D_2h) or 24 h (D_24h) relative to leaves that were not exposed to drought stress (D_0h). DEGs were selected based on a cut-off of *P*-adjust < 0.05 and |log_2_FC| ≥ 1. (**a)** Number of up- and down-regulated differentially expressed genes (DEGs) between D_0h and D_2h, and between D_0h and D_24h. (**b)** Venn analysis of DEGs between D_0h vs. D_2h and D_0h vs. D_24h. (**c**) Top 20 KEGG pathways enriched by DEGs at 0 h vs. 2 h. (**d**) Top 20 KEGG pathways enriched by DEGs at 0 h vs. 24 h

The correlation analysis of all transcriptome samples showed that D_2h samples were strongly and positively correlated with D_0h and D_24h samples (Fig. S1a). Principal component analysis (PCA) was used to reduce the dimension of the data and to visualize the relationship among samples. The first principal component (PC1) and the second principal component (PC2) explained 66.48% and 15.96% of the total variation, respectively (Fig. S1b). Compared with control (D_0h), a total of 5033 DEGs were identified at 2 h (D_2h) of simulated drought stress, with 2299 significantly up-regulated genes and 2734 significantly down-regulated genes. A total of 8159 DEGs were obtained from 0 h vs. 24 h comparisons, consisting of 3724 significantly up-regulated genes and 4435 significantly down-regulated genes (Fig. 1a). For overlap analysis, we identified 4206 DEGs in both the D_2h samples and the D_24h samples (Fig. 1b).

**Fig. 2:**
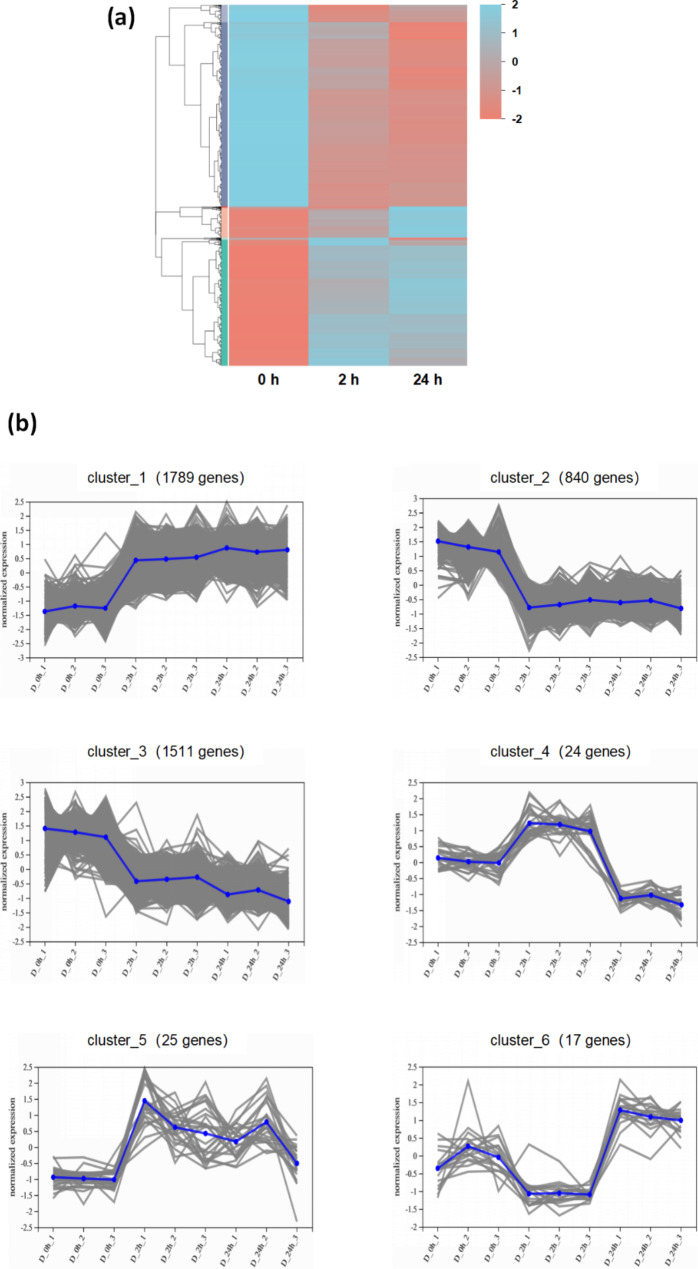
Hierarchical cluster analysis of common DEGs in *C. equisetifolia* leaves that were exposed to drought stress for different times. DEGs were selected based on a cut-off of *P*-adjust < 0.05 and |log_2_FC| ≥ 1. **(a)** Hierarchical cluster analysis of common DEGs in D_0 h vs. D_2 h and D_0 h vs. D_24 h. Red represents down-regulation and blue represents up-regulation of gene expression. Expression values were Z-scaled (log_10_ FPKM). **(b)** The expression patterns of six clusters. The blue line indicates the average expression values of all genes in the gene set

To explore the function of DEGs, we performed KEGG enrichment analysis (Fig. 1c, d). Relative to D_0h samples, there were three highly representative KEGG pathways in both D_2h and D_24h samples, which were “plant hormone signal transduction” (*P*-adjust_(2 h vs. 0 h)_ = 0.000, *P*-adjust_(24 h vs. 0 h)_ = 0.000), “plant-pathogen interaction” (*P*-adjust_(2 h vs. 0 h)_ = 0.007, *P*-adjust_(24 h vs. 0 h)_ = 0.042), and “MAPK signaling pathway” (*P*-adjust_(2 h vs. 0 h)_ = 0.000, *P*-adjust_(24 h vs. 0 h)_ = 0.000). The numbers of DEGs in each pathway mentioned above were greater in D_24h samples than in D_2h samples. For example, only 75 DEGs were assigned to “plant hormone signal transduction” in D_2h, while 107 DEGs were assigned to “plant hormone signal transduction” in D_24h (Table S3 ). In total, 4206 genes were regarded as DEGs in both pairwise comparisons (0 h vs. 2 h and 0 h vs. 24 h) under drought stress treatment. The expression patterns of the common 4206 DEGs were visualized with a heatmap (Fig. 2a). The result showed that these DEGs could be divided into two main groups: one group had the highest transcript levels in the control (D_0h), while the other group had the highest transcript levels under drought treatment. To understand the general trend of these DEGs, we performed *k*-means clustering analysis and obtained 6 clusters (Fig. 2b). The top three clusters with the largest number of DEGs were cluster 1 (1789 genes), cluster 3 (1511 genes), and cluster 2 (840 genes) (Fig. 2b). In cluster 1, where transcript levels of 1789 genes were up-regulated after drought stress treatment (Fig. 2b), and the top two pathways were “MAPK signaling pathway” and “plant hormone signal transduction” (Table S4 ). In cluster 2, all of the DEGs exhibited a dramatic decrease during the treatment (Fig. 2b), and the top two pathways in cluster 2 were “plant hormone signal transduction” and “flavonoid biosynthesis” (Table S4 ).

To verify the accuracy of transcriptome data during drought stress, six DEGs from pathways of plant hormone signal transduction, phenylpropanoid and flavonoid biosynthesis were selected for qRT-PCR. The results of RNA-seq showed that three DEGs were up-regulated while three DEGs were down-regulated (Fig. S2). The expression trends of six DEGs were consistent with the transcriptomic data, which verifies its reliability and accuracy.

### Metabolomics of C. equisetifolia in response to drought stress

**Fig. 3:**
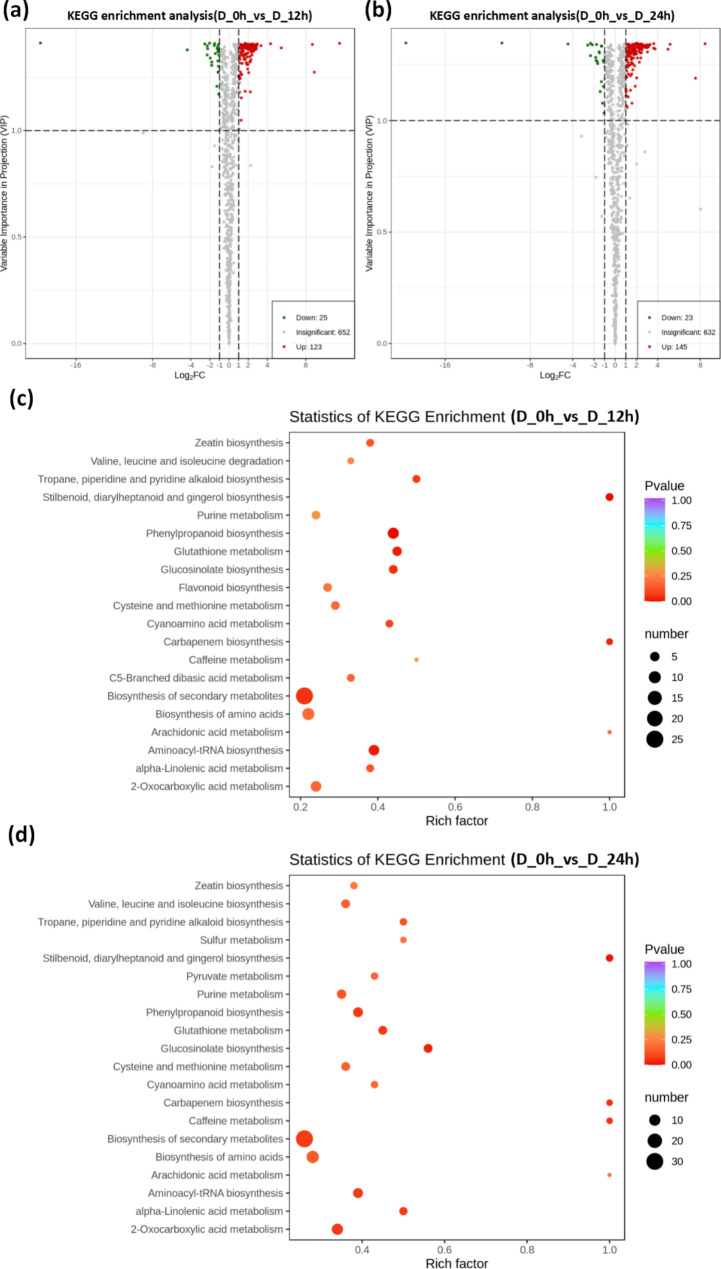
Differentially accumulated metabolites (DAMs) at different times in *C. equisetifolia* under drought stress. DAMs were selected based on a cut-off of VIP ≥ 1, *P*-value < 0.05, fold-change ≥ 2, and fold-change ≤ 0.5. Volcano plot of DAMs in **(a)** D_0h vs. D_12h and in **(b)** D_0h vs. D_24h. **(c)** Top 20 enriched pathways for DAMs of D_0h vs. D_12h. **(d)** Top 20 enriched pathways for DAMs of D_0h vs. D_24h

To understand the changes of metabolites under drought stress in *C. equisetifolia*, we conducted a wide analysis of metabolites on D_0h, D_12h, and D_24h samples, and identified the differentially accumulated metabolites (DAMs). In comparison to the control (D_0h), 148 DAMs were found in D_12h samples, with 123 significantly up-regulated and 25 significantly down-regulated metabolites (Fig. 3a). A total of 168 metabolites were identified as DAMs in D_24h samples, consisting of 145 significantly up-regulated and 23 significantly down-regulated metabolites (Fig. 3b). To reveal the pathways of DAMs, we performed a KEGG pathway enrichment analysis. In D_12h samples, the top five pathways were “biosynthesis of secondary metabolites”, “biosynthesis of amino acids”, “phenylpropanoid biosynthesis”, “aminoacyl-tRNA biosynthesis”, and “2-oxocarboxylic acid metabolism” (Fig. 3c). However, the top five pathways in D_24h samples were “biosynthesis of secondary metabolites”, “biosynthesis of amino acids”, “2-oxocarboxylic acid metabolism”, “ABC transporters”, “aminoacyl-tRNA biosynthesis”, and “phenylpropanoid biosynthesis” (Fig. 3d). The contents of many compounds increased significantly under drought stress, and these compounds were mainly involved in amino acid biosynthesis, phenylpropanoid biosynthesis, and flavonoid biosynthesis. In the metabolic pathway of amino acid biosynthesis, the contents of methionine, leucine, isoleucine, proline, and phenylalanine increased significantly.

### Plant hormone signal transduction pathways involved in the response to drought stress

**Fig. 4:**
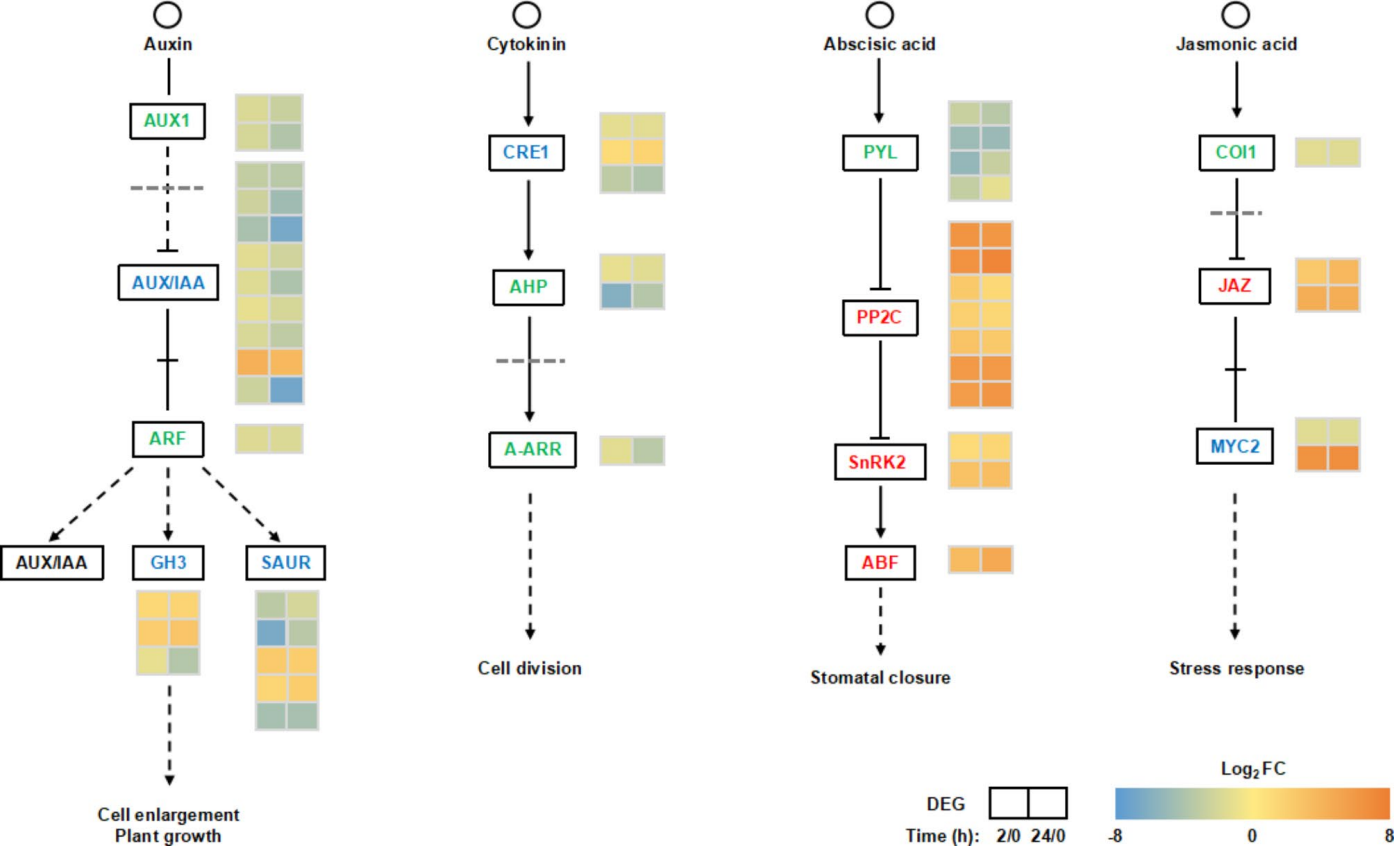
DEGs involved in plant hormone signal transduction at different times in *C. equisetifolia* under drought stress. The original figure of plant hormone signal transduction pathway was cited from the Kanehisa laboratories [69]. DEGs were selected based on a cut-off of *P*-adjust < 0.05 and |log_2_FC| ≥ 1. The two boxes represent log_2_FC (D_2h / D_0h) and log_2_FC (D_24h / D_0h), respectively. Red text indicates significantly up-regulated DEGs, green text indicates significantly down-regulated DEGs, and blue text indicates both significantly up-regulated and down-regulated DEGs. AUX1: Auxin-Resistant1. AUX/IAA: Auxin/Indole-3-Acetic Acid. ARF: auxin response factor. GH3: Gretchen hagen 3. SAUR: Small auxin up RNA. CRE1: Cytokinin receptor (1) AHP: histidine phosphotransfer proteins. A-ARR: type-A response regulator. PYL: pyrabactin resistance-like. PP2C: phosphatase 2 C. SnRK2: ABA-activated SNF1-related protein kinases (2) ABF: ABA-responsive element binding factor. COI1: coronatine insensitive 1. JAZ: jasmonate ZIM-domain. MYC2: transcription factor

**Fig. 5:**
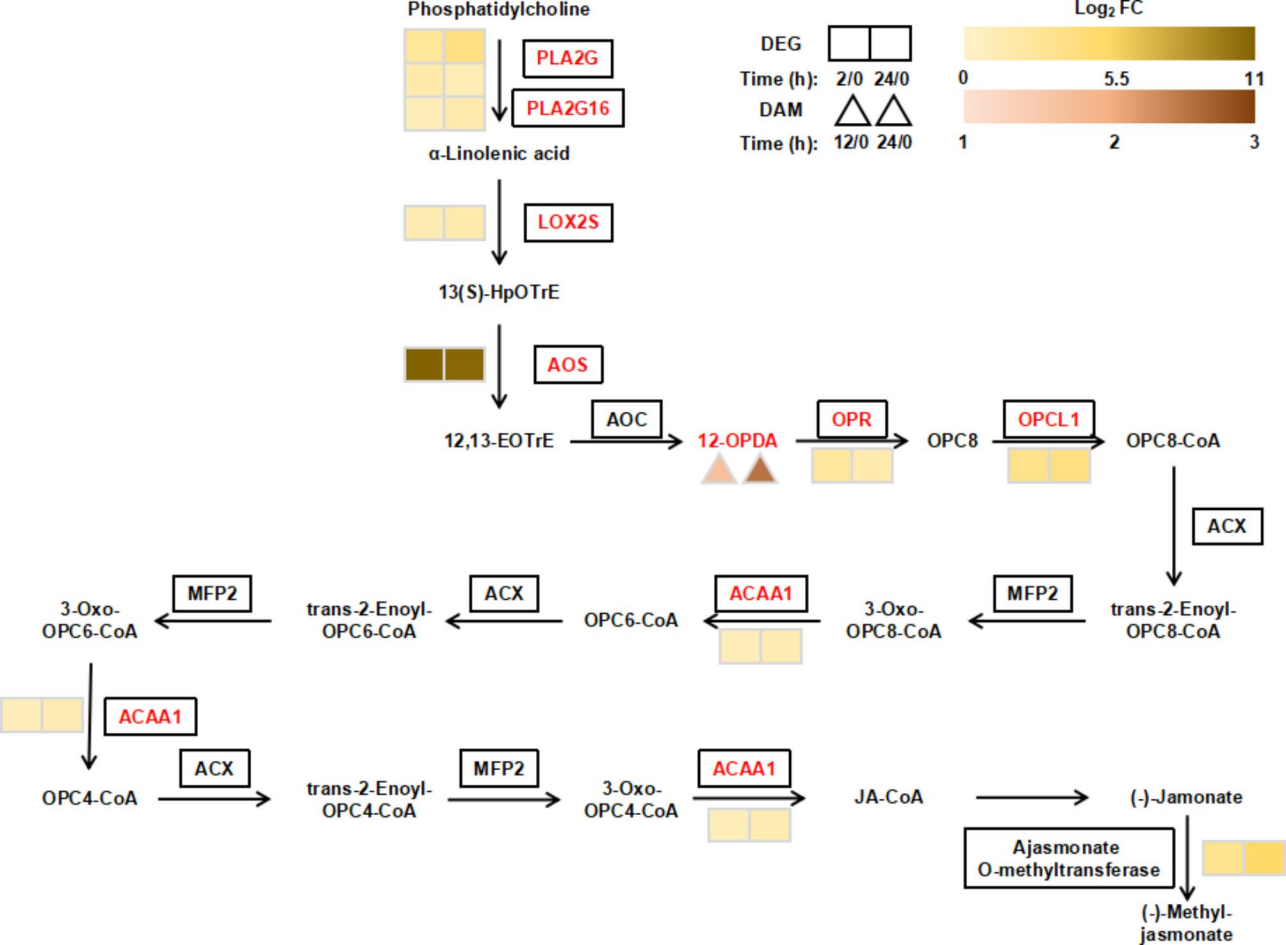
DEGs and DAMs in the jasmonic acid (JA) biosynthetic pathway at different times in *C. equisetifolia* under drought stress. The original figure of JA biosynthetic pathway was cited from the Kanehisa laboratories [70, 71, 72]. DEGs were selected based on a cut-off of *P*-adjust < 0.05 and |log_2_FC| ≥ 1. DAMs were selected based on a cut-off of VIP ≥ 1, *P*-value < 0.05, fold-change ≥ 2, and fold-change ≤ 0.5. The two boxes represent log_2_FC (D_2h / D_0h) and log_2_FC (D_24h / D_0h) of DEGs, respectively. The two triangles represent log_2_FC (D_2h / D_0h) and log_2_FC (D_24h / D_0h) of DAMs, respectively. Red text represents significantly up-regulated DEGs/DAMs. AOS: allene oxide synthase. OPR: 12-oxo-phytodienoic acid reductase. OPCL1: OPC-8:0 CoA Ligase1. ACAA1: acetyl-CoA acyltransferase 1

The “plant hormone signal transduction” pathway was highly represented in the KEGG enrichment analysis (Fig. 1c, d). To analyze the relationship between plant hormones and the drought stress response in *C. equisetifolia*, we investigated the genes related to the plant hormone signal transduction under drought stress using RNA-seq data. Four plant hormone signal transduction pathways, including ABA, auxin, cytokinin, and JA, were involved in the drought stress response (Fig. 4). For the ABA signal transduction pathway, 4 DEGs encoding ABA receptors pyrabactin resistance/pyrabactin resistance-like (PRY/PYL) proteins were significantly down-regulated, while the 7 DEGs encoding type 2 C protein phosphatase (PP2C), 2 DEGs encoding ABA-activated SNF1-related protein kinases 2 (SnRK2), and 1 DEG encoding ABA-responsive element binding factor (ABF) were significantly up-regulated, suggesting the importance of ABA in the drought stress response. In the JA signal transduction pathway, 1 DEG encoding coronatine insensitive 1 (COI1, significantly down-regulated), 2 DEGs encoding jasmonate ZIM-domain (JAZ, significantly up-regulated), and 2 DEGs encoding MYC2 (1 significantly down-regulated and 1 significantly up-regulated) were expressed significantly under drought stress (Fig. 4). In addition, the 8 DEGs involved in JA biosynthesis were significantly up-regulated, including 3 DEGs encoding PLA2G/PLA2G16, 1 DEG encoding LOX2S, 1 DEG encoding 12-oxo-phytodienoic acid reductase (OPR), 1 DEG encoding OPC-8:0 CoA Ligase1 (OPCL1), 1 DEG encoding ACAA1, and 1 DEG encoding jasmonate O-methyltransferase (Fig. 5).

### Secondary metabolism involved in the response to drought stress

**Fig. 6:**
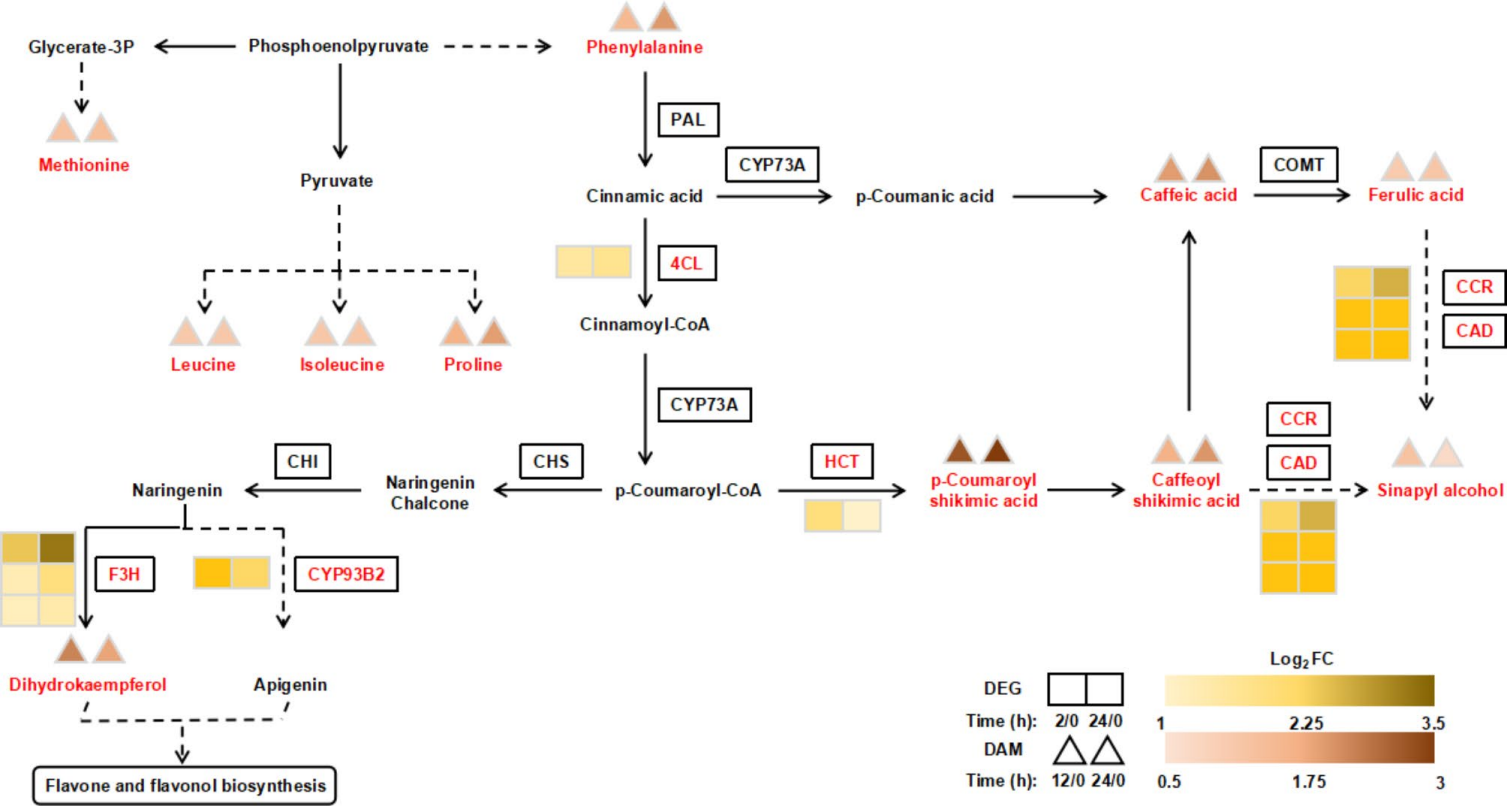
DEGs and DAMs in amino acid, phenylpropanoid, and flavonoid biosynthetic pathways at different times under drought stress. The original figures of amino acid, phenylpropanoid, and flavonoid biosynthetic pathways were cited from the Kanehisa laboratories [70, 71, 72]. DEGs were selected based on a cut-off of *P*-adjust < 0.05 and |log_2_FC| ≥ 1. DAMs were selected based on a cut-off of VIP ≥ 1, *P*-value < 0.05, fold-change ≥ 2, and fold-change ≤ 0.5. The two boxes represent log_2_FC (D_2h / D_0h) and log_2_FC (D_24h / D_0h) of DEGs, respectively. The two triangles represent log_2_FC (D_2h / D_0h) and log_2_FC (D_24h / D_0h) of DAMs, respectively. Red text represents significantly up-regulated DEGs/DAMs. 4CL: 4-coumarate: coenzyme A ligase. HCT: hydroxycinnamoyl transferase. CCR: cinnamoyl-coA reductase. CAD: cinnamoyl alcohol dehydrogenase. F3H: Flavanone 3-hydroxylase

Drought treatments (both 12 and 24 h) significantly increased the accumulation of methionine, leucine, isoleucine, and proline in *C. equisetifolia*. The significant accumulation of its compounds in phenylpropanoid and flavonoid biosynthesis was consistent with the DEGs whose expression levels were significantly up-regulated in these pathways. For example, the shikimate *O*-hydroxycinnamoyl transferase gene *HCT* was up-regulated more than 1.98-fold at 2 h and 1.02-fold at 24 h after drought stress in *C. equisetifolia* (Fig. 6). The product of this enzyme, namely p-coumaryl shikimic acid, was significantly up-regulated after drought stress (Fig. 6). The significant increase of dihydrokaempferol and sinapyl alcohol is likely the result of the significant up-regulation of these DEGs under drought stress (Fig. 6).

## Discussion

### Plant hormone signal transduction pathways in response to drought stress

Many studies of plants have shown that ABA- and JA-mediated signal transduction pathways are related to the response to drought stress [38–40]. It has been reported that the ABA and JA signaling pathways of *C. equisetifolia* and *Casuarina glauca* were activated under salt stress [41, 42]. ABA that accumulates under drought stress can activate specific serine / threonine kinase family SnRK2, which can phosphorylate downstream transcription factors and activate ABA response genes [43, 44]. In this study, there were 2 DEGs encoding SnRK2 significantly up-regulated in *C. equisetifolia* subjected to both 2 and 24 h of drought stress. PYL proteins are ABA receptors that are involved in the response of plants to ABA [45]. Overexpression of *PYLs* in *Arabidopsis* can promote its response to ABA, but some *PYLs* are down-regulated after exogenous ABA treatment [46, 47]. The expression pattern of *PYLs* in *Arabidopsis* can be explained by negative feedback regulation [46, 48]. Our results showed that 4 *PYLs* were significantly down-regulated after drought treatment, which was consistent with the previous studies. Researchers have also reported that drought stress activated downstream transcription factors involved in ABA response, and the activated transcription factors may in return regulate the expression of *PYLs* and *PP2Cs* [45]. In the current study, ABA response mediated downregulation of receptor PYLs, upregulation of negative regulator PP2Cs, and upregulation of the downstream transcription factor ABF. The negative feedback can reduce the adverse effects caused by ABA accumulation, which may help to increase the adaptability of *C. equisetifolia* to drought stress.

JAs not only regulate plant growth and development, but also improve plant tolerance to environmental stress via the JA signaling pathway [49]. JA accumulation induced by stress activates downstream transcription factors and then activates regulatory genes in response to stress [50]. The expression of *COI1* in *Poa pratensis* leaves was recently found to be inhibited at the initial stage of drought stress [51]. We found that *COI1* expression was significantly down-regulated in *C. equisetifolia*, suggesting that the JA signaling pathway was involved in the response to drought. Previous studies have shown that overexpression of *JAZ6* in *Oryza sativa* and JAZs in *Poa pratensis* can increase the tolerance of those plants to mannitol and drought stress [51, 52]. There were 2 up-regulated DEGs encoding JAZs in treated *C. equisetifolia* branchlets, which may help improve the drought tolerance of the plant. It is reported that the *MYC2* transcription level in *Arabidopsis* decreased significantly under drought stress [53]. Our results showed that the expression of 1 DEG encoding MYC2 was significantly down regulated, which was consistent with the results of previous studies. These results suggested that the plant hormones might be important in the drought stress response of *C. equisetifolia*.

### The JA biosynthesis pathway in response to drought stress

JA can increase the tolerance of plants to water stress by regulating stomata, scavenging reactive oxygen species, and altering root development [50]. Previous studies have shown that continuous drought stress promoted the up-regulation of the JA biosynthesis genes *OPR*, *ACX*, and *ACAA1* in *P. pratensis* [51] and *AOS* and *OPR* in *Cicer arietinum* [54]. The JA biosynthesis gene *ACAA1* is related to peroxisome, and the up-regulation of its expression may help remove reactive oxygen species produced by drought stress [51]. Our results showed that the expressions of the JA biosynthesis genes *OPR*, *AOS*, and *ACAA1* were significantly up-regulated, which is consistent with previous studies [51, 54]. One of the substrates for the synthesis of JA, 12-OPDA, is also a signal molecule with overlapping and different functions from JA [55, 56]. Previous studies have shown that the accumulation of 12-OPDA reduces stomatal apertures and increases plant drought tolerance [55]. We also found that 12-OPDA of *C. equisetifolia* accumulated significantly under drought stress, which is consistent with previous studies. In *C. equisetifolia*, the significantly up-regulated genes and significantly increased metabolites in JA biosynthesis pathway may improve drought tolerance in this species.

### Secondary metabolism in response to drought stress

Amino acids are osmotic regulators that maintain and stabilize cell structure and that usually accumulate in plants in response to osmotic stress [57]. Previous studies have shown that plants (*O. sativa, Zea mays*, and *Arabidopsis*) accumulate a large number of amino acids (such as proline, leucine, tryptophan, isoleucine, and histidine) to increase drought tolerance [18, 58, 59]. In addition, *C. glauca* accumulates ornithine and proline under salt stress to regulate osmotic pressure [60]. In this study, the contents of proline, leucine, isoleucine, methionine, and phenylalanine were significantly increased in response to drought stress, indicating that amino acid metabolism was involved in the response of *C. equisetifolia* to drought stress.

Plants respond to oxidative damage induced by osmotic stress by accumulating active oxygen scavengers such as phenols and flavonoids [61]. Many studies have shown that the accumulation of phenols and flavonoids in plants can improve drought tolerance [62, 63]. It has been reported that the biosynthesis pathway of secondary metabolites of *C. glauca* was activated under salt stress, and the increase of flavonoid content alleviated oxidative damage [28, 64–66]. Our results showed that the pathways of phenylpropanoid and flavonoid biosynthesis in *C. equisetifolia* respond to drought stress at the transcriptomic and metabolomic levels. The accumulation of one flavonoid (dihydrokaempferol) and five phenolic acids (p-coumaroyl shikimic acid, caffeoyl shikimic acid, caffeic acid, ferulic acid, and sinapyl alcohol) in the current study was significantly increased under drought stress, which was consistent with the results of previous studies. Previous studies have shown that overexpression of *F3H* (an important gene in the flavonoid biosynthesis pathway) and *4CL* (4-coumaric acid CoA ligase, a branch point in the phenylpropanoid biosynthesis pathway) can increase the drought tolerance of plants [67–69]. We therefore suspect that flavonoids, phenols, and related genes may be involved in the response of *C. equisetifolia* to drought stress.

## Conclusion

We analyzed the transcript and metabolic profiles of *C. equisetifolia* branchlets to increase our understanding of the mechanisms underlying the plant’s responses to drought stress. Pathway analysis showed that DEGs under drought stress were related to ABA and JA signal transduction pathways, phenylpropanoid biosynthesis, and flavonoid biosynthesis. Drought stress not only induced the expression of genes related to JA biosynthesis but also increased the accumulation of flavonoids and phenols and the expression of related genes. Our results also showed that drought stress increased the accumulation of amino acids, such as leucine, isoleucine, proline, methionine, and phenylalanine, which could help *C. equisetifolia* regulate osmotic pressure and maintain cell structure under drought stress. These results increase our understanding of how *C. equisetifolia* tolerates or resists drought stress at both transcriptional and metabolic levels and provide reference data for the management and restoration of regional vegetation.

## Electronic supplementary material

Below is the link to the electronic supplementary material.

Supplementary Material 1

## Data Availability

The sequenced raw reads generated in this study have been submitted to the National Center for Biotechnology Information (NCBI) with BioProject ID: PRJNA902157 (https://dataview.ncbi.nlm.nih.gov/object/PRJNA902157?reviewer=fa84revdsbtmdef8f97ahav6q5).
