# Supplementary material for: Transcriptome and metabolome analysis reveals key genes and secondary metabolites of Casuarina equisetifolia ssp. incana in response to drought stress
Source: BMC Plant Biol. 2023 Apr 18;23:200. doi: 10.1186/s12870-023-04206-x (PMC10111710; doi:10.1186/s12870-023-04206-x)
Supplement: Supplementary file 1 — Supplementary Material 1 [file 12870_2023_4206_MOESM1_ESM.docx]

**Supporting information**


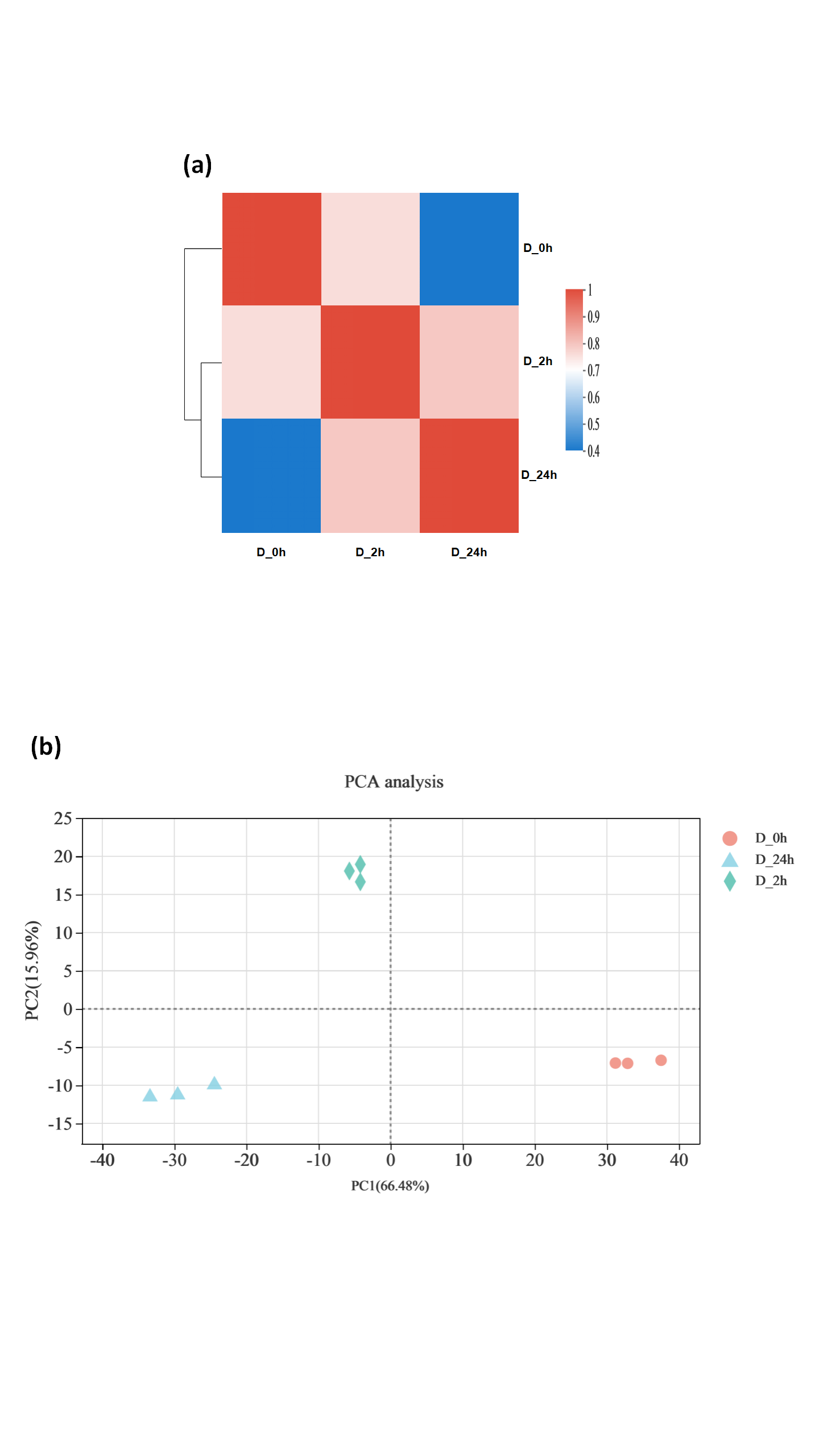
 **Figure S1. (a)** Correlation among the samples. The heat map is based on Pearson correlation coefficient, complete linkage clustering method, and Euclidean distance algorithm. **(b)** PCA plot of the transcriptome of D_0h, D_2h, and D_24h samples.


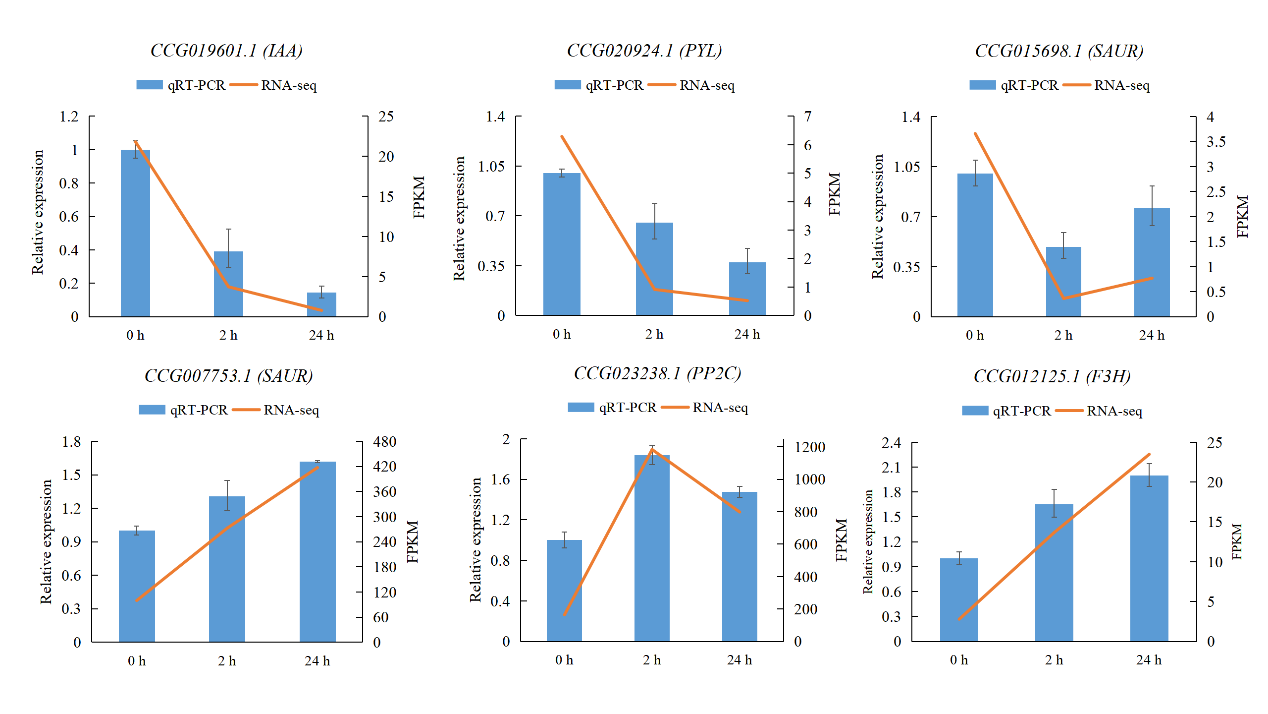


**Figure S2.** Quantitative real-time PCR (qRT-PCR) validation of six genes from plant hormone signal transduction, phenylpropanoid, and flavonoid biosynthetic pathways. *IAA,* *Indole-3-Acetic Acid; PYL,* *pyrabactin resistance-like; SAUR, Small auxin up RNA; PP2C, phosphatase 2C; F3H, Flavanone 3-hydroxylase.* The expression level in the RNA-seq is based on the average value of FPKM at each stage.

**Table S1.** Primers for qRT-PCR.

| Primer name | Sequence |
| --- | --- |
| EF1α-F | TGGCATGTACTTGAGGATCACA |
| EF1α-R | TGCATTGATGGCACGAAGC |
| UBI-F | GAAAAACCATAACCTTGGAAGTTG |
| UBI-R | GATTCCTTTTGGATGTTGTAATCC |
| CCG019601.1(IAA)-F | AACCAGCAGCAGAAGAAGAG |
| CCG019601.1(IAA)*-*R | GTAGCCCGTGTACGTCATTAG |
| *CCG020924.1(PYL)*-F | GATCACCGAGACCCATGTTAC |
| *CCG020924.1(PYL)-*R | GTACCATACGATCACCTCCAAC |
| *CCG015698.1(SAUR)*-F | CGAGATGGGTCTCACAATTCC |
| *CCG015698.1(SAUR)-*R | CAAGTCTACGCCTGCCTAATC |
| *CCG007753.1(SAUR)*-F | CTGTTCGGTGAGCTTCTAAGG |
| *CCG007753.1(SAUR)-*R | GGTTCTGGACTCTCTCGAAATC |
| *CCG023238.1(PP2C)*-F | TCGGTCTCTCTCCATCATCTT |
| *CCG023238.1(PP2C)-*R | TCTCTCCGCCTTCCACATA |
| *CCG012125.1(F3H)*-F | GGAGCAGTACAGTGAGAGATTG |
| *CCG012125.1(F3H)-*R | CTTGGTCAAAGCCTCCTTCT |

**Table S2. Summary of RNA-seq.**

| Sample | Clean reads | Q30 (%) | GC content (%) | Total mapped reads (Percentage in clean reads) | Multiple mapped reads (Percentage in clean reads) | Uniquely mapped reads (Percentage in clean reads) |
| --- | --- | --- | --- | --- | --- | --- |
| D_0h_1 | 42,132,106 | 95.23 | 47.88 | 39,331,362  (93.35%) | 1,918,071  (4.55%) | 37,413,291  (88.8%) |
| D_0h_2 | 43,776,378 | 95.16 | 47.75 | 40,869,740  (93.36%) | 1,605,138  (3.67%) | 39,264,602  (89.69%) |
| D_0h_3 | 45,892,522 | 95.41 | 47.74 | 42,767,680  (93.19%) | 1,561,285  (3.4%) | 41,206,395  (89.79%) |
| D_2h_1 | 45,856,492 | 95.18 | 47.48 | 39,331,362  (93.35%) | 2,859,831  (6.24%) | 39,791,931  (86.77%) |
| D_2h_2 | 41,804,404 | 95.08 | 47.54 | 40,869,740  (93.36%) | 1,494,862  (3.58%) | 37,575,606  (89.88%) |
| D_2h_3 | 43,084,806 | 95.04 | 47.64 | 42,767,680  (93.19%) | 1,723,642  (4.0%) | 38,296,666  (88.89%) |
| D_24h_1 | 47,073,278 | 93.80 | 47.33 | 42,159,359  (89.56%) | 2,654,838  (5.64%) | 39,504,521  (83.92%) |
| D_24h_2 | 49,195,586 | 93.74 | 47.15 | 44,479,994  (90.41%) | 2,877,047  (5.85%) | 41,602,947  (84.57%) |
| D_24h_3 | 44,544,988 | 93.39 | 47.61 | 40,930,512  (91.89%) | 4,236,663  (9.51%) | 36,693,849  (82.37%) |

**Table S3.** Top 4 KEGG pathways of DEGs in D_0h vs. D_12h and D_0h vs. D_24h.

|  | Number | Pathway id | Description | P-adjust |
| --- | --- | --- | --- | --- |
| D_2h vs. D_0h | 75 | map04075 | Plant hormone signal transduction | 0.000 |
|  | 56 | map04626 | Plant-pathogen interaction | 0.007 |
|  | 50 | map00500 | Starch and sucrose metabolism | 0.007 |
|  | 48 | map04016 | MAPK signaling pathway - plant | 0.000 |
| D_24h vs. D_0h | 107 | map04075 | Plant hormone signal transduction | 0.000 |
|  | 96 | map00940 | Phenylpropanoid biosynthesis | 0.003 |
|  | 79 | map04626 | Plant-pathogen interaction | 0.042 |
|  | 70 | map04016 | MAPK signaling pathway - plant | 0.000 |

**Table S4.** Top 3 KEGG pathways of cluster_1 and cluster_2.

|  | Number | Pathway id | Description | P-adjust |
| --- | --- | --- | --- | --- |
| cluster_1 | 29 | map04016 | MAPK signaling pathway - plant | 0.000 |
|  | 26 | map04075 | plant hormone signal transduction | 0.039 |
| cluster_2 | 15 | map04075 | plant hormone signal transduction | 0.017 |
|  | 9 | map00941 | flavonoid biosynthesis | 0.017 |
